# Hyperlactatemia and pulmonary gas exchange at rest and during exercise: lessons from patients on metformin

**DOI:** 10.1007/s00421-026-06213-w

**Published:** 2026-04-10

**Authors:** Philippe Haouzi, William W. Stringer, Jonathan Mccully, Bohdan M. Pichurko

**Affiliations:** 1 Department of Pulmonary Medicine, Cleveland Clinic, 9500 Euclid Avenue, Cleveland, OH 44195 USA; 2 Lundquist Institute for Biomedical Innovation at Harbor UCLA Medical Center, 1124 West Carson Street, Torrance, CA 90509 USA; 3 Division of Respiratory and Critical Care, Physiology and Medicine, Harbor-UCLA Medical Center, 1000 West Carson Street, Torrance, CA 90509 USA

**Keywords:** Metformin, Hyperlactatemia, Gas-exchange threshold, Lactic acid threshold, Respiratory exchange ratio

## Abstract

What effect does elevated resting hyperlactatemia have on the response to a moderate level of exercise and on the determination of the gas-exchange threshold (GET)? We first identified 163 diabetic patients treated with metformin, a medication that has been shown to produce, in a significant proportion of patients, an innocuous high resting hyperlactatemia and who underwent cardio-pulmonary exercise testing at the Cleveland Clinic. We found that 102 patients had resting [La] < 2 mM (Group 0); 37 had [La] between 2 and 3 mM (Group 1); 12 had [La] between 3 and 3.5 mM (Group 2); and 12 had [La] > 3.5 mM (Group 3). We examined relationships between resting [La], minute ventilation, pulmonary gas exchange, pH, and arterial blood gases at rest and during exercise (Welch’s test, and Cohen’s d to estimate effect size). None of the baseline data exhibited an effect size consistent with a clinically meaningful confounder. Group 3 had significantly lower HCO₃⁻ (22.5 ± 2.3 mM) than Group 0 (24.2 ± 2.4 mM, *p* < 0.01). ${\dot{\text {V}}}$E was also significantly higher in Group 3. Despite elevated lactate in Group 3 at rest, the respiratory exchange ratio (RER) averaged 0.85 ± 0.12 and did not differ from the other groups. GET could be identified based on the ${\dot{\text {V}}}$O₂/${\dot{\text {V}}}$CO₂ relationship in all groups. Finally, the ventilatory and gas exchange responses to exercise showed no differences between groups. An elevated blood lactate concentration is insufficient, by itself, to reproduce the gas-exchange signature of supra-threshold exercise or to disrupt GET determination.

## Introduction

One of the most important tenets of the analysis of a non-invasive cardiopulmonary exercise testing (CPET) relies on the determination the level of exercise at which lactate concentration increases in the blood, referred as the lactic acid threshold (LT) (Poole et al. 2021). LT is a submaximal indicator of fitness and a good predictor of the severity of chronic pulmonary diseases, pulmonary hypertension, or heart diseases (ATS/ACCP 2003; Sietsema et al. 2021; Balady et al. 2010; Agostoni and Guazzi 2017; Buono et al. 2019; Guazzi et al. 2017). There has been a long debate on the physiological meaning and the mechanisms behind the determination of LT (Poole et al. 2021; Brooks 1985, 2018, 2021; Koike et al. 1985; Wasserman and Koike 1992; Wasserman et al. 1994; Katz and Sahlin 1985; Peronnet and Aguilaniu 2006). As developed by the “Wasserman group” (Wasserman et al. 1973), increased lactate accumulation in blood and tissues during exercise leads to a metabolic acidosis, which in turn causes the transformation of HCO3^−^ into free CO₂. The release of CO₂ from HCO3^−^, in addition to the CO₂ produced in the Krebs cycle and by the pyruvate reaction, leads to an increase in CO_2_ ouput (${\dot{\text {V}}}$CO₂) that exceeds oxygen uptake (${\dot{\text {V}}}$O₂), and thus to an increase in ${\dot{\text {V}}}$CO₂/${\dot{\text {V}}}$O₂ ratio, the respiratory equivalent ratio (RER). Plotting ${\dot{\text {V}}}$CO₂ on the Y axis and ${\dot{\text {V}}}$O₂ on the X axis (the “V-slope method”) allows the identification of LT (Beaver et al. 1986). The change in the slope of the ${\dot{\text {V}}}$CO₂/${\dot{\text {V}}}$O₂ relationship, also refereed to as the “inflection point”, is therefore considered as a non-invasive surrogate for an elevation of lactate concentration in the blood (Wasserman et al. 1990). We will use the term gas exchange threshold (GET) when referring to the determination of LT via this method.

The question addressed in the current study is the following: can a high lactate concentration at rest and during moderate exercise reproduce the characteristics of a supra-LT exercise? These characteristics include disproportionate rise in ventilation (high ${\dot{\text {V}}}$E/${\dot{\text {V}}}$O₂), increased CO₂ output from buffering out of proportion of O₂ (high RER), rising end tidal O₂ partial pressure (P_ET_O₂), falling end tidal CO_2_ partial pressure (P_ET_CO₂).

To test this hypothesis, we revisited an observation reported by Davis et al. (2001), based on the Fremantle Diabetes study, who showed that about one third of diabetic patients on metformin displayed a chronic and innocuous resting elevated lactate ranging from 2 to 5 mM. This picture should be firmly distinguished from the life-threatening hyperlactatemia and acidosis produced by metformin toxicity, thought to be related to an acute and potentially fatal blockade of mitochondrial complex I (Mizock and Falk 1992; DeFronzo et al. 2016). In contrast, such a chronic increase in resting lactate concentration (up to 5 mM) appears to be an incidental finding in these ambulatory populations. The mechanisms of La accumulation in these patients are not fully understood and could be explained by an alteration (inhibition) of gluconeogenesis (Madiraju et al. 2018) (decreasing La utilization) or, less likely, by a partial impediment of the electron chain (increasing La production) (Fontaine et al. 2018; LaMoia and Shulman 2021; Luengo et al. 2014).

We took advantage of the large data set accumulated in our institution to identify patients on metformin who performed a non-invasive CPET and in whom blood lactate concentration ([La-]) and GET were determined. Our goal was to establish the relationship between resting [La] and pulmonary gas exchange responses at rest and during exercise. We sought to determine whether an elevated blood lactate concentration at rest, when “uncoupled from muscle contraction-induced hyperlactatemia ”, can reproduce the pulmonary gas-exchange signature of supra-threshold exercise or interfere with non-invasive determination of the gas-exchange threshold.

## Methods

### Study design

We used a list of 2808 de-identified adult patients (age ≥ 18 years) who performed a CPET in the pulmonary function laboratory of the Department of Pulmonary Medicine at the Cleveland Clinic between 2010 and 2023. Patients at the Cleveland Clinic sign a general consent that includes participation in research. The present study was reviewed by our IRB and was found not to require IRB approval (de-entified patients). We found 184 patients in this cohort who were diabetic and treated by metformin. The search included any of the following medications: “biguanide”, “metformin”, “glucophage”, “fortamet”, “Riomet”, “Axpinet”, “Diagemet”, “Glucient”, “Metabet”, “Glumetza”. Indications for CPET were shortness of breath or pre-operative evaluation.

Of the 184 patients on these medications, 163 had a determination of whole blood lactate levels and arterial blood gases (ABG). Both were measured from the same blood sample, on the same device, and on the same day as the CPET (ABL800 Flex 800 (through 2022) or ABL 90 (since 2022), Radiometer, Medical ApS, Brønshøj, Denmark).

Blood sampled from a radial artery was measured prior to (20 min before) exercise and at exercise termination (within one minute following exercise cessation). Of note, blood was obtained through direct puncture (no arterial line was placed).

In addition, baseline spirometry (MedGraphics Platinum Elite™ body plethysmograph system, MGC Diagnostics, Saint Paul, MN, USA) was obtained 20–30 min before each CPET study; 69 patients had an echocardiography within 3 months preceding the test, and 99 of these 163 patients had a blood creatinine level determined within one month of testing.

The CPETs were performed according to ATS recommendations (American Thoracic 2003). Our pulmonary function testing laboratory provides CPET and blood gas and lactate measurements for the entire Cleveland Clinic health network and operates under standardized verification and quality-assurance procedures. These include routine electronic quality control testing, automated sensor calibration against known standards, participation in external proficiency testing, and strict pre-analytical requirements. All quality control, calibration, and maintenance procedures are systematically documented, consistent with established practice in clinical exercise physiology.

Each subject performed a ramp-like exercise test protocol on an electrically braked cycle ergometer (Corival Lode CPET, Lode, Groningen, The Netherlands) to exhaustion. The increment in work rate was determined before each test in keeping with the patient’s level of fitness, so peak exercise was achieved within 10–15 min. The subjects breathed through a low-dead-space face mask connected to a pneumotachograph with sampling lines to measure inspired and expired gas concentrations. Minute ventilation, oxygen uptake (${\dot{\text {V}}}$O₂), and carbon dioxide output (${\dot{\text {V}}}$CO₂) were obtained breath-by-breath (MedGraphics Ultima CPX system, MGC Diagnostics, Saint Paul, MN, USA). GET was determined based on the ${\dot{\text {V}}}$O₂-to-${\dot{\text {V}}}$CO₂ relationship (V-slope method) (Wasserman et al. 1973, 1990). Transcutaneous O₂ saturation (TcSO₂), blood pressure, and ECG were monitored throughout the test and recovery. All patients were requested to have a light lunch or breakfast before the study.

The CPET data were acquired entirely within a guideline-based clinical framework. Peak ${\dot{\text {V}}}$O₂ was therefore determined according to the joint American Thoracic Society/American College of Chest Physicians recommendations and represents the highest oxygen uptake achieved during a symptom-limited test. Test termination was based on patient symptoms, safety considerations, and clinical judgment rather than exhaustion of “physiological reserve”. Consequently, peak ${\dot{\text {V}}}$O₂ maximum HR or lactate should not be interpreted as equivalent to or a ‘true’ maximal test, but corresponded to the peak exercise reach by each patients.

Data were filtered, using as an 8 breath-moving average. Five to eight breath-moving average is standard in clinical practice to reduce “noise” in the data and is recommended for graphical, real-time, or near-real-time viewing. Calibrations were performed before each test. Of note, no test could be performed unless a proper calibration of flow (linearity, gain), along with the determination of the gain and the time response of the O₂ and CO₂ analyzers have been successfully performed. GET was determined automatically by the Medgraphic software, then it was validated by the physician (authors of this paper) performing the CPET interpretation. This was done by visual analysis of the breaking point ${\dot{\text {V}}}$O₂/${\dot{\text {V}}}$CO₂ relationship and by evaluating the coherence of this value with the changes in ${\dot{\text {V}}}$E/${\dot{\text {V}}}$CO₂ and RER. In some patients, the level at which the subjects stopped exercise was too low to be able to properly identify GET (see result section).

### Data analysis

**Fig. 1:**
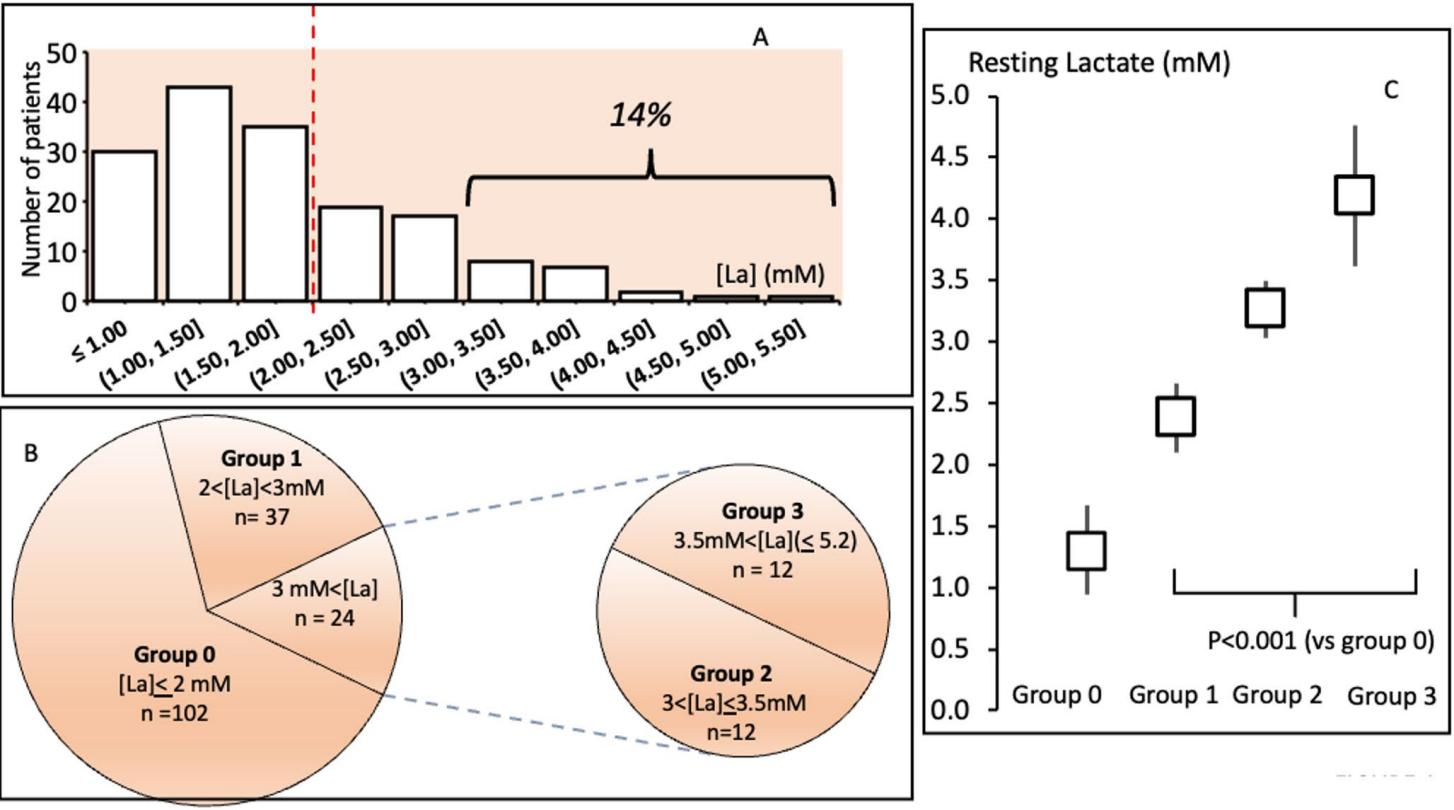
Frequency distribution of blood lactate concentration in our population of diabetic patients

Patients were classified according to resting blood lactate level prior to CPET testing: Group 0—normal lactate ([La] < 2 mM) and patients with [La] > 2 mM. The second group of patients was then subdivided into borderline Group 1 ([La] 2–3 mM), moderately elevated lactate Group 2 ([La] 3–3.5 mM), and further elevated lactate Group 3 ([La] > 3.5 mM), as displayed in Fig. 1. Minute ventilation, pulmonary gas exchange, and heart rate were determined at rest, at GET, and at peak exercise. Arterial pH, [H+], [HCO3^−^], base excess (BE), arterial partial pressure of O_2_ (PaO₂), arterial partial pressure of CO_2_ (PaCO₂), and arterial O_2_ saturation (SaO₂), O₂ content, and concentration in hemoglobin (Hb) were determined at rest and at peak exercise. Finally, we identified patients in Group 0 who reached peak exercise lactate levels similar to those in Group 3 at rest and compared their resting and exercise data, as above.

Because group sizes were unequal and variance heterogeneity was anticipated across resting lactate group, we deliberately avoided classical one-way ANOVA and pooled-variance *t* tests, which rely on assumptions that are rarely satisfied in retrospective clinical CPET datasets. Instead, global group comparisons were performed using Welch’s one-way ANOVA, which provides valid control of Type I error under unequal sample sizes and unequal variances. For pairwise comparisons, Welch’s *t*-test was similarly preferred over Student’s *t*-test. In parallel, effect sizes were reported using Cohen’s *d* to quantify the standardized magnitude of group differences independently of sample size and statistical significance, and Hedges’ *g* was provided as a small-sample bias–corrected estimate for comparisons involving the smaller lactate groups. Effect sizes were interpreted using conventional thresholds: Cohen’s *d* ≈ 0.2 (small), 0.5 (moderate), 0.8 (large).

Reporting effect sizes alongside *p* values is particularly important in exploratory physiological analyses, where absence of statistical significance may reflect limited power rather than true equivalence, and where interpretation of physiological relevance depends on the magnitude and direction of effects. This combined approach—Welch-based inference with standardized effect sizes—allows transparent evaluation of both statistical robustness and physiological meaning.

## Results

### Resting lactate concentrations in patients on metformin

The demographic and physiologic characteristics of the 163 patients are shown in Table 1. As shown in Fig. 1 and 102 patients (62.5%, Group 0) had a resting [La] < 2 mM (1.30 ± 0.37 mM), whereas 61 patients had [La] > 2 mM. Among these 61 patients, 37 had [La] between 2 and 3 mM (2.38 ± 0.29 mM, Group 1), 12 had [La] between 3 and 3.5 mM (3.18 ± 0.18 mM, Group 2), and 12 had [La] > 3.5 mM (4.04 ± 0.56 mM, Group 3), with a maximal resting level of 5.2 mM. The frequency distribution is shown in Fig. 1. Of note, in our entire CPET population not receiving metformin, lactate values > 3 mM were rare (33 of 2196 patients, 1.5%) compared with ~ 15% in metformin-treated patients (*P* < 0.0001), indicating a strong association between metformin therapy and resting hyperlactatemia.

**Table 1:** Comparison of patients’ characteristics according to resting lactate concentration

	Group 0 2 *≤* La		Group1 2 < La *≤* 3		Group 2 3 > La *≤* 3.5		Group3 La > 3.5		Welch ANOVA *p*	Hedges’ g vs. G0 (G1/G2/G3)	Welch *p* vs. G0 (G1/G2/G3)
	(*n* = 102)		(*n* = 37)		(*n* = 12)		(*n* = 12)				
	Mean	SD	Mean	SD	Mean	SD	Mean	SD			
Lactate (mM)	1.30	0.37	2.38	0.29	3.18	0.18	4.04	0.56			
Age	61	13	61	12	59	19	66	10	0.42	0.00/−0.14/0.39	1.00/0.73/0.13
men/women ratio	0.60		0.46		0.25		0.50				
Height (cm)	171	10	171	11	**162**	9	170	12	**0.00015**	0.00/**−0.90/**−0.10	1.00/**0.006**/0.79
Weight (kg)	99	20	98	21	98	22	98	22	0.94	−0.05/−0.05/−0.05	0.80/0.88/0.88
BMI (kg/m^2^)	34	6	33	9	37	8	35	2	**0.0044**	−0.14/0.48/0.17	0.53/0.23/0.23
β blockers (% patients)	27		21		19		28				
EF (%)	59	9	61	4	63	4	60	10	**0.026**	0.25/0.46/0.11	0.24/0.45/0.11
	*n* = 40		*n* = 13		*n* = 5		*n* = 6				
Creatinine (mg/l)	1.06	0.44	1.00	0.37	0.88	0.15	0.96	0.10	0.076	−0.14/−0.43/−0.24	0.43/0.006/0.059
	*n* = 58		*n* = 22		*n* = 7		*n* = 6				
HBAIC (%)	7.17	1.13	7.54	1.68	7.00	1.17	6.91	1.30	0.23	0.28/−0.15/−0.22	0.22/0.64/0.52
Hb (g/100 ml)	13.4	2.2	13.0	1.5	12.5	1.7	13.1	1.3	**0.032**	−0.17/−0.38/−0.11	0.30/0.15/0.59

Group differences were tested using Welch one-way ANOVA (unequal variances). Pairwise comparisons vs. Group 0 were performed using Welch t tests. Effect sizes are reported as Cohen’s *d* after Hedges’ *g* correction. Note that n was lower for the ejection fraction (EF) and creatine levels that were not determined in every patient. Difference across lactate groups and clinical variables showed no consistent effect-size pattern relative to Group 0, with isolated moderate effects confined to small subgroups (notably Group 2), supporting robust group comparability. Significant p and Hedges’ *g* ≥ 0.8 are shown in bold

### Demographic and baseline clinical characteristics

As shown in Table 1, baseline demographic and clinical variables (age, weight, BMI, sex, cardiac ejection fraction, creatinine, pulmonary function indices, SaO₂, PaO₂, and HbA1c) were comparable across lactate groups. Effect sizes were uniformly small (*d* < 0.3) for age, BMI, cardiac function, renal indices, hemoglobin, and oxygenation. The only notable difference was a lower mean height in Group 2, which did not follow a monotonic relationship with lactate concentration. Thus, the groups were well balanced, and no baseline variable demonstrated an effect size consistent with a clinically meaningful confounder.

### Resting ventilatory, pulmonary gas exchange, and arterial blood gas parameters

As shown in Table 2, global Welch ANOVA across lactate groups identified differences in breathing frequency, PaCO₂, PaO₂, and bicarbonate concentration. Pairwise exploratory comparisons versus Group 0 showed that these effects were driven primarily by Group 3, which exhibited higher minute ventilation and breathing frequency, associated with slightly higher PaO₂ and lower PaCO₂, along with lower bicarbonate and base excess.

**Table 2:** Resting *data* are shown as mean ± SD

	G0		G1		G2		G3		Welch ANOVA *p*	Hedges’ g vs. G0 (G1/G2/G3)	Welch *p* vs. G0 (G1/G2/G3)
	Mean	SD	Mean	SD	Mean	SD	Mean	SD			
${\dot{\text {V}}}$O₂ (L min⁻¹)	0.33	0.09	0.32	0.07	0.29	0.07	0.37	0.19	0.16	−0.12/−0.45/0.38	0.49/0.09/0.49
${\dot{\text {V}}}$O₂/kg (ml kg⁻¹ min⁻¹)	3.4	0.8	3.3	0.6	3.1	0.5	3.8	1.6	0.10	−0.13/−0.38/0.44	0.43/0.08/0.41
RER	0.85	0.10	0.85	0.11	0.82	0.10	0.85	0.12	0.33	0.00/−0.30/0.00	1.00/0.34/1.00
Heart rate (bpm)	83	16	89	15	87	15	87	11	0.14	0.38/0.25/0.26	0.045/0.40/0.28
${\dot{\text {V}}}$E (L min⁻¹)	12.4	3.4	12.8	3.4	11.6	3.2	14.5	4.2	0.16	0.12/−0.24/0.60	0.54/0.43/0.12
${\dot{\text {V}}}$E/${\dot{\text {V}}}$O₂	38	7	40	6	40	8	42	12	0.31	0.29/0.28/0.52	0.10/0.42/0.28
BF (min⁻¹)	17	5	19	5	18	4	**24**	9	**0.021**	0.40/0.20/**1.26**	0.041/0.44/**0.022**
pH	7.41	0.03	7.41	0.03	7.40	0.03	7.40	0.03	0.22	0.00/−0.33/−0.33	1.00/0.29/0.29
[H⁺] (nM)	38.7	2.8	38.8	2.4	39.7	2.9	29.8	2.5	0.29	0.04/0.35/0.39	0.84/0.28/0.18
PaCO₂ (mmHg)	39	4	38	4	38	6	37	4	**0.014**	−0.25/−0.24/−0.50	0.20/0.58/0.12
PaO₂ (mmHg)	83	9	86	11	79	15	**90**	16	**0.0026**	0.31/−0.41/**0.70**	0.14/0.38/0.16
HCO₃⁻ (mM)	24.2	2.4	23.3	2.4	23.3	2.8	**22.5**	2.3	**0.018**	−0.37/−0.37/**−0.71**	0.055/0.30/**0.030**
Base excess (mM)	0.0	2.4	−0.8	2.4	−1.0	2.5	−1.7	2.2	0.34	−0.33/−0.41/−0.71	0.087/0.21/0.025
SaO₂ (%)	94	2	94	2	93	3	94	1	0.43	0.00/−0.47/0.00	1.00/0.28/1.00

Global group differences were assessed using Welch one-way ANOVA. Pairwise comparisons versus Group 0 were performed using Welch’s t-test. Effect sizes are reported as Cohen’s *d* and Hedges’ *g*. RER = respiratory exchange ratio. Welch ANOVA was significant for some variables, and pairwise comparisons versus Group 0 showed that these effects were driven primarily by Group 3, which exhibited higher ventilation and breathing frequency, lower bicarbonate and base excess. See text for more details. RER, pH, and${\dot{\text {V}}}$CO₂-related indices did not differ across groups, indicating preserved gas-exchange behavior despite resting hyperlactatemia. Significant *p* and Hedges’ *g* > 0.8 are shown in bold

Importantly, RER, pH, and ${\dot{\text {V}}}$CO₂-related indices did not differ across groups, and effect sizes were uniformly small. Thus, despite mild acid–base shifts, resting hyperlactatemia did not produce a pattern consistent with supra-threshold physiology.

### Response to exercise

#### Responses at GET

**Fig. 2:**
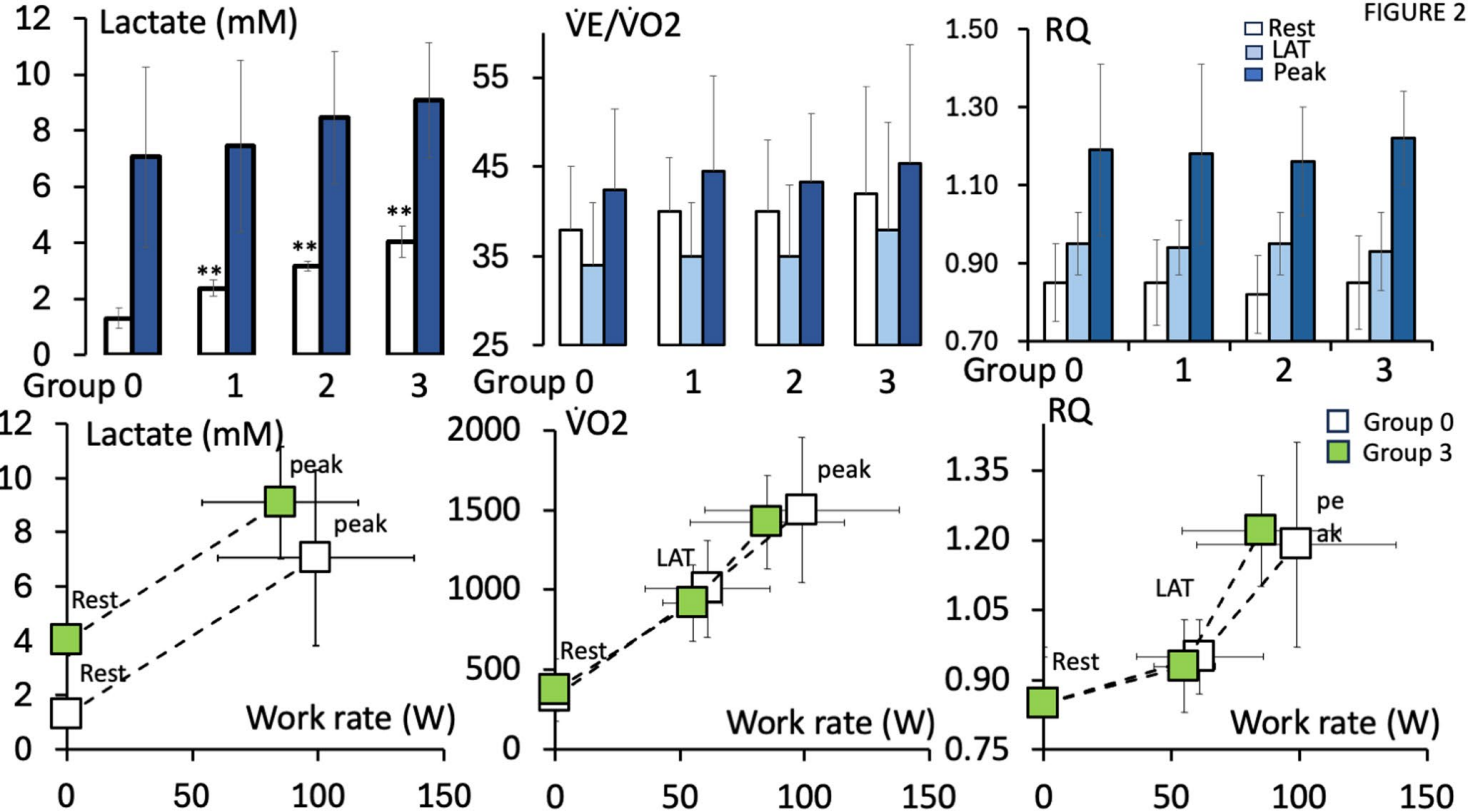
Upper panels: Panel A: lactate concentrations at rest and at peak exercise in the four groups. Panel B: ${\dot{\text {V}}}$E/${\dot{\text {V}}}$O₂ ratio at rest, GET, and peak exercise in the four groups. Panel C: RER at rest, GET, and peak exercise in the four groups. Note that, despite differences in lactate at rest and even at peak exercise, criteria for LT determination based on ${\dot{\text {V}}}$CO₂/${\dot{\text {V}}}$O₂ (GET) were present in all groups (see Table 3 and text). Lower panels show the relationships between work rate (x-axis) and La (D), ${\dot{\text {V}}}$O₂ (E), and RER (F) (y-axis) in Groups 0 and 3. Despite different lactate levels at rest between Group 0 and Group 3, responses to exercise were indistinguishable. **Significantly different from Group 0

GET could be identified in 139 of 163 patients. In the remaining 24 patients, exercise was terminated before reaching GET. At GET, no variable demonstrated a statistically detectable group effect by global Welch ANOVA. All pairwise comparisons versus Group 0 were non-significant, with small effect sizes (|g| generally < 0.30) (Table 3; Fig. 2). This remained true after combining Groups 2 and 3. Thus, elevated resting lactate did not interfere with identification of the gas-exchange threshold.

**Table 3:** Mean ± SD of ventilatory, pulmonary gas exchange, and arterial blood gas parameters at GET and peak exercise according to resting lactate level

	Group 0	Group 1	Group 2	Group 3	Welch ANOVA *p*	Hedges’ g vs. G0 (G1/G2/G3)
*GET*						
Work rate (W)	61 ± 25	54 ± 23	57 ± 23	55 ± 12	0.52	−0.28/−0.16/−0.29
${\dot{\text {V}}}$O₂ (L min⁻¹)	1.01 ± 0.30	0.93 ± 0.27	0.94 ± 0.23	0.92 ± 0.24	0.41	−0.28/−0.26/−0.31
${\dot{\text {V}}}$O₂/kg (ml kg⁻¹ min⁻¹)	10.5 ± 3.6	10.0 ± 2.4	9.9 ± 3.0	9.9 ± 1.4	0.67	−0.15/−0.17/−0.21
RER	0.95 ± 0.08	0.94 ± 0.07	0.95 ± 0.08	0.93 ± 0.10	0.74	−0.13/0.00/−0.24
${\dot{\text {V}}}$E/${\dot{\text {V}}}$O₂	34 ± 10	35 ± 9	35 ± 7	38 ± 6	0.29	0.11/0.13/0.43
${\dot{\text {V}}}$E (L min⁻¹)	33.4 ± 10.2	32.8 ± 8.6	31.7 ± 7.3	33.5 ± 6.5	0.88	−0.06/−0.18/0.01
Heart rate (bpm)	108 ± 19	110 ± 21	115 ± 25	106 ± 15	0.63	0.10/0.33/−0.11
*Peak exercise*						
Work rate (W)	99 ± 39	87 ± 39	72 ± 29	85 ± 31	0.11	−0.31/−0.74/−0.36
Heart rate (bpm)	127 ± 23	132 ± 21	133 ± 27	122 ± 19	0.46	0.22/0.24/−0.22
Lactate (mM)	7.06 ± 3.23	7.46 ± 3.07	8.47 ± 2.38	9.10 ± 2.06	0.18	0.12/0.46/0.67
Δ-lactate (mM)	5.72 ± 3.34	5.06 ± 2.80	4.92 ± 2.45	5.31 ± 2.15	0.69	−0.21/−0.26/−0.12
${\dot{\text {V}}}$E (L min⁻¹)	63.7 ± 23.3	60.3 ± 20.2	51.7 ± 15.5	60.6 ± 13.8	0.22	−0.15/−0.58/−0.14
${\dot{\text {V}}}$O₂ (L min⁻¹)	1.50 ± 0.46	1.38 ± 0.47	1.18 ± 0.29	1.42 ± 0.29	0.10	−0.26/−0.77/−0.18
${\dot{\text {V}}}$O₂/kg (ml kg⁻¹ min⁻¹)	15.6 ± 5.1	14.5 ± 4.2	12.5 ± 3.5	14.5 ± 3.0	0.18	−0.23/−0.63/−0.24
${\dot{\text {V}}}$E/${\dot{\text {V}}}$O₂	42 ± 9	44 ± 11	43 ± 8	45 ± 13	0.57	0.20/0.12/0.27
RER	1.19 ± 0.22	1.18 ± 0.23	1.16 ± 0.14	1.22 ± 0.12	0.83	−0.04/−0.15/0.15
pH	7.36 ± 0.05	7.38 ± 0.03	7.36 ± 0.04	7.36 ± 0.03	0.41	0.44/0.00/0.00
[H⁺] (nM)	43.9 ± 4.9	42.5 ± 3.5	43.8 ± 3.6	44.0 ± 3.3	0.58	−0.32/−0.02/0.02
PaCO₂ (mmHg)	35 ± 5.6	34 ± 5	38 ± 8	33 ± 5	0.27	−0.19/0.45/−0.36
PaO₂ (mmHg)	94 ± 15	95 ± 14	87 ± 16	97 ± 14	0.49	0.07/−0.44/0.20
HCO₃⁻ (mM)	19.6 ± 3.7	19.4 ± 3.2	20.5 ± 3.9	18.2 ± 2.4	0.51	−0.06/0.23/−0.42
BE (mM)	−5.0 ± 3.8	−4.9 ± 3.2	−4.2 ± 3.7	−6.4 ± 2.4	0.47	0.03/0.21/−0.41
SaO₂ (%)	93 ± 2	94 ± 3	93 ± 3	95 ± 2	0.32	0.37/0.00/0.63

Global group differences were assessed using Welch one-way ANOVA. Effect sizes are reported as Cohen’s *d* test with Hedges’ *g* (small sample) correction. Across resting lactate groups, ventilatory, gas-exchange, and arterial blood gas variables at GET and peak exercise showed no systematic differences by Welch ANOVA and only small, non-directional effect sizes versus Group 0, supporting similar exercise responses despite elevated baseline lactate. RER = respiratory exchange ratio

#### Peak exercise

For peak exercise variables, global Welch ANOVA was non-significant, and no pairwise comparison versus Group 0 reached statistical significance when variance heterogeneity and small sample sizes were accounted for. Effect sizes (Cohen’s d) were uniformly small to moderate and non-monotonic.

More specifically, ventilatory, gas-exchange, and arterial blood gas responses were similar across resting lactate groups, with no systematic group differences and only small, non-monotonic effect sizes versus Group 0. These findings indicate preserved exercise response despite elevated baseline lactate.

When Groups 2 and 3 were combined, global Welch ANOVA identified differences only for ${\dot{\text {V}}}$O₂ and ${\dot{\text {V}}}$O₂/kg at peak exercise. Pairwise Welch tests versus Group 0 confirmed lower peak oxygen uptake, with moderate effect sizes (Hedges’ g ≈ − 0.6 to − 0.7). No significant differences were observed for ventilatory ratios, RER, or arterial acid–base variables.

${\dot{\text {V}}}$O₂, work rate, and RER at GET and peak exercise were not different between groups. There were no significant differences in pH, HCO₃⁻, minute ventilation, RER, or Δ lactate at GET or peak exercise (Table 3). The main exercise results are summarized in Fig. 2.

**Fig. 3:**
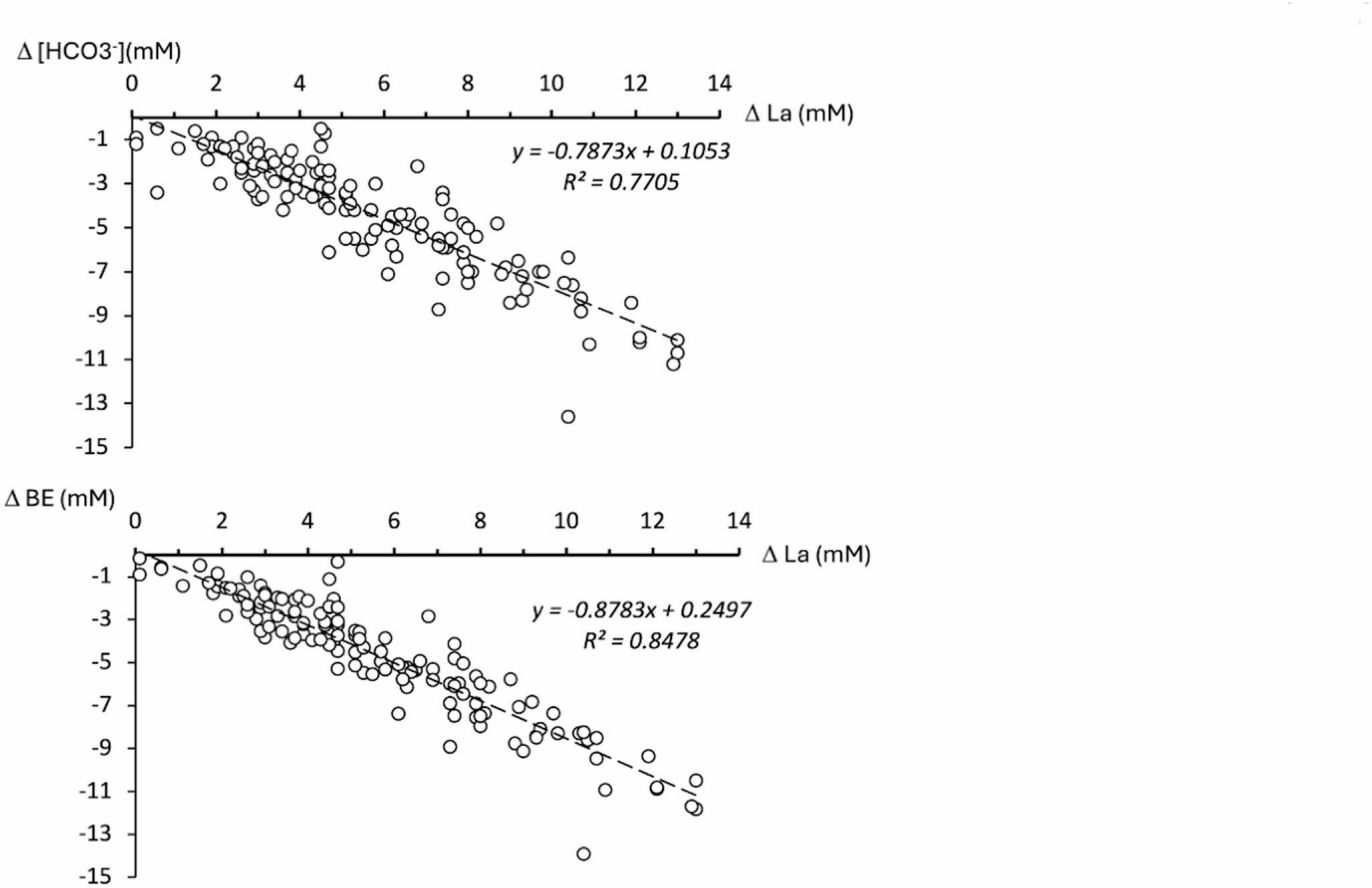
Regression analysis between Δ[HCO₃⁻] and ΔBE vs. Δ lactate (exercise minus rest), showing a significant correlation between these variables and lactate

Finally, as illustrated in Fig. 3, there was a strong inverse correlation between ΔHCO₃⁻, ΔBE, and Δ lactate (exercise minus rest). The slopes were − 0.79 for ΔHCO₃⁻ and − 0.88 for ΔBE, indicating tight coupling between lactate accumulation during exercise and bicarbonate buffering across physiological states.

### Comparison of two groups with similar blood lactate concentrations attained either at peak exercise in Group 0 or at rest (Group 3)

Fifteen patients in Group 0 reached a lactate concentration between 3.5 and 4.2 mM at peak exercise (Table 4), similar to the resting lactate level observed in Group 3. These 2 groups with nearly identical lactate concentrations (~ 4.1 mM) exhibited markedly different physiological responses depending on whether lactate elevation occurred at rest or during exercise. Patients in Group 0, who reached this level during exercise, demonstrated a significantly higher RER (≈ 1.10) and higher hydrogen ion concentration, consistent with exercise-induced metabolic acidosis accompanied by excess CO₂ production.

**Table 4:** Comparison of the physiological responses (mean ± SD) in two groups with comparable blood lactate concentrations (~ 4 mM) achieved either during peak exercise (from Group 0) or at rest (Group 3)

	Group 0 3.5 < La (peak ex) < 5.2 (*n* = 15)	Group 3 (*n* = 12)	*p* (Welch)	Cohen’s d (95% CI)
	Mean ± SD (Peak Ex)	Mean ± SD (Rest)		
Lactate (mM)	4.13 ± 0.46	4.04 ± 0.56	0.64	0.18 (− 0.56, 0.92)
RER	**1.10 ± 0.12**	**0.85 ± 0.12**	**< 0.001**	**2.08 (1.05**,** 3.11)**
pH	7.38 ± 0.02	7.40 ± 0.03	0.06	−0.79 (− 1.62, 0.04)
[H⁺] (nM)	**41.3 ± 1.8 **	**39.5 ± 2.0**	**0.02**	**0.97 (0.12**,** 1.81)**
PaCO₂ (mmHg)	36 ± 3	36 ± 5	0.99	0.00 (− 0.74, 0.74)
PaO₂ (mmHg)	92 ± 11	90 ± 16	0.70	0.15 (− 0.59, 0.89)
HCO₃⁻ (mM)	**20.8 ± 2.3**	**22.5 ± 2.3**	**0.04**	**−0.74 (− 1.57**,** − 0.01)**
Base excess (mM)	**−3.5 ± 2.3**	**−1.7 ± 2.2**	**0.03**	**−0.80 (− 1.64**,** − 0.04)**
${\dot{\text {V}}}$E (L min^−1^)	13.7 ± 1.7	13.1 ± 1.3	0.33	0.40 (− 0.35, 1.15)
SaO₂ (%)	94 ± 2	94 ± 1	0.99	0.00 (− 0.74, 0.74)

Despite similar lactate levels, peak-exercise lactatemia was associated with a markedly higher respiratory quotient, greater hydrogen ion concentration, and more negative base excess, consistent with a high rate of proton generation and bicarbonate buffering producing excess CO₂. In contrast, resting hyperlactatemia showed a normal respiratory quotient and attenuated acid–base changes, indicating that lactate concentration alone does not determine CO₂ excess or the ventilatory response. Significant *p* and large Hedges’ *g* > 0.8 are shown in bold

In contrast, Group 3 patients with comparable lactate levels at rest showed a normal RER (≈ 0.85), lower [H⁺], and less negative base excess. PaCO₂, PaO₂, Hb concentration, and arterial oxygen saturation were indistinguishable between groups.

In summary, iso-lactatemia at rest was associated with a different acid–base and gas-exchange signatures than during exercise.

## Discussion

In this retrospective analysis of metformin-treated patients undergoing cardiopulmonary exercise testing, we show that chronic, incidental resting hyperlactatemia (up to ~ 5 mM) is associated with a mild, compensated metabolic acidosis at rest but does not increase ${\dot{\text {V}}}$CO₂ out of proportion to ${\dot{\text {V}}}$O₂ (RER). Despite elevated resting lactate levels, ventilatory control, pulmonary gas exchange, and acid–base responses during moderate and peak exercise were preserved, and whenever a gas-exchange threshold could be reliably identified using standard non-invasive methods, it was not affected by the level of resting lactate. The response to exercise was essentially indistinguishable between Group 0 (normal lactate at rest) and Group 3 (high lactate at rest). These findings indicate that resting hyperlactatemia per se does not mimic supra-threshold exercise physiology and does not confound CPET-based determination of the lactate (gas-exchange) threshold. These findings further suggest that increased blood lactate concentration, per se, does not always drive a non-metabolic CO₂ “signal,” and that more complex mechanisms govern the emergence of supra-threshold pulmonary gas-exchange behavior.

### High resting lactate in diabetic patients treated with metformin

Our first finding was that, akin to the results reported by Davis et al. (2001), a proportion of patients chronically treated with metformin display resting lactate concentrations between ~ 3 and 5 mM, as illustrated in Fig. 1. We found that ~ 14-15% of metformin-treated patients had a lactate level above 3 mmol, and half of these patients had a lactate above 3.5 mM. Davis et al. (2001) measured lactate in fasting subjects from a community-based cohort. In contrast, our CPET patients were instructed to consume a light meal before testing. therefore, meal volume and composition were uncontrolled variables that could have influenced baseline lactate concentration (Leija et al. 2024). Yet, this variability is intrinsic to real-world clinical testing and does not alter the central observation that elevated resting lactate can exist without proportional excess CO₂ production or altered gas-exchange behavior.

Unlike the Fremantle Diabetes Study, patients in the present cohort were referred for CPET because of unexplained dyspnea, underlying cardiopulmonary disease (low FEV1 or FVC, reduced ejection fraction), or pre-operative evaluation. Despite these differences, the distribution of blood lactate concentrations was remarkably similar to that reported by Davis et al. (2001). Given that lactate is a gluconeogenic precursor, inhibition of this pathway by metformin remains a plausible mechanism underlying chronic resting hyperlactatemia in these patients.

No relationship was found between resting lactate concentration and conditions potentially leading to tissue hypoxia, including arterial oxygenation, cardiac function, or hemoglobin concentration. In the broader CPET population (*n* = 2196), the incidence of resting lactate above 3 mM was low (33 of 2196 patients, 1.51%), compared with 15% in metformin-treated patients. Thus, elevated lactate was more than tenfold more prevalent in patients receiving metformin.

### Acid–base changes associated with hyperlactatemia at rest and during exercise: high resting lactate does not affect RER or exercise responses

The physiological literature has long debated whether lactate accumulation is the primary driver of exercise-induced metabolic acidosis (Poole et al. 2021; Sietsema et al. 2021; Brooks 1985; Katz and Sahlin 1985; Wasserman et al. 1967, 1973; Beaver et al. 1986; Wasserman 1994, 1999; Koike et al. 1994). Alternative mechanisms, including ATP hydrolysis–related proton generation, have been proposed to account for acid–base changes during exercise (Gladden 2008).

Despite nearly identical blood lactate concentrations (~ 4 mM), physiological signatures differed strikingly depending on whether lactate accumulation occurred acutely during exercise or chronically at rest (Table 4). As shown of this table, when lactate reached ~ 4 mM during exercise, it was accompanied by elevated RER, increased [H⁺], and more negative base excess—consistent with rapid proton generation and bicarbonate buffering with excess CO₂ production. In contrast, the same lactate concentration present at rest was associated with a normal RER and attenuated acid–base disturbance. These findings indicate that lactate concentration per se does not drive excess CO₂ output; changes associated with the rate of lactate accumulation drive the gas exchange response.

Indeed, as displayed in Fig. 3, we found a significant negative correlation between the increase in concentration in La and the reduction in bicarbonate, which indicates the bicarbonate buffering of protons and rate of CO₂ “production” are coupled with the rate of lactate accumulation (typically estimated by its concentration), but not the rate of lactate generation. As we still have a very incomplete understanding of the mechanisms underlying metformin-associated hyperlactatemia (DeFronzo et al. 2016, Madiraju et al. 2018; Fontaine et al. 2018; LaMoia and Shulman 2021; Luengo et al. 2014), any mechanistic interpretations would remain quite speculative. Nevertheless, this finding suggests that the assumed fixed stoichiometric relationship between lactate concentration, proton load and bicarbonate buffering in exercise does not hold (or has been compensated) under these steady state resting conditions in this population. Although our study does not provide any mechanism(s) on why a chronic lactate accumulation at rest differs from exercise, the present findings identify a serious limitation of an oversimplification of the “classical” Wasserman model: the equimolar coupling between lactate concentration and proton generation, implicit in this model, may not generalize to chronic hyperlactatemia. The change in lactate concentrations produced by exercise (not the absolute value) appears to continue to be detected by gas exchange measures.

### Limitations of the study, theoretical and practical considerations

The present analyses were conducted on a retrospective, clinically derived dataset characterized by unequal group sizes across resting lactate groups. Under these conditions, classical fixed-effects ANOVA and pooled-variance t-tests could exhibit inflated type I error rates. Global group comparisons were therefore performed using Welch’s one-way analysis of variance. Welch ANOVA has been shown to maintain appropriate error control making it the preferred test for group comparisons in observational and clinical physiology datasets. Because the primary aim of this study was to evaluate whether elevated resting lactate concentration was associated with systematic alterations in ventilatory or gas-exchange behavior—rather than to establish equivalence or superiority of specific groups—pairwise comparisons were conducted in an exploratory framework without formal correction for multiple testing. In addition, standardized effect sizes were reported using Cohen’s *d*, with Hedges’ *g* for small sample involving smaller lactate groups. Effect sizes were interpreted in conjunction with global test results to avoid overreliance on *p* values.

The retrospective nature of the study and the fact that the cohort was unidentified prevented the control of various aspects of the study using an heterogenous population. However, our primary question was whether resting hyperlactatemia disrupts the behavior and interpretability of gas-exchange indices under routine clinical testing conditions, not whether absolute threshold levels differ across protocol-dependent metrics. Accordingly, protocol heterogeneity was treated as a fundamental feature of real-world clinical CPET rather than a confounder requiring statistical adjustment. Finally, mixed-effects or repeated-measures models were not employed because the study design does not involve repeated measurements of the same physiological variable across conditions within individuals.

## Conclusions

Our results shows that diabetic patients treated with metformin with elevated resting lactate exhibit a compensated metabolic acidosis but without an accompanying increase in ${\dot{\text {V}}}$CO₂/${\dot{\text {V}}}$O₂ ratio. Resting hyperlactatemia shifted lactate upward during exercise but did not alter ventilatory or gas-exchange behavior below the lactate threshold, nor did it interfere with threshold identification. These findings suggest that exercise intensity–dependent processes, rather than elevated lactate concentration per se, govern the emergence of supra-threshold pulmonary gas-exchange behavior.
